# Epidural Inflammatory Pseudotumor in the Cervical Spine: A Case Report of a Bernese Mountain Dog

**DOI:** 10.3390/ani15071049

**Published:** 2025-04-04

**Authors:** Yoshiyuki Inoue, Rie Kitoh, Moe Satoh, Yuki Yoshigae, Kazumi Nibe, Kazuyuki Uchida, Satoru Matsunaga

**Affiliations:** 1 Japan Animal Referral Medical Center, Kawasaki 213-0032, Japan; rie.kitou@jarmec.jp (R.K.); d5109@jarmec.jp (M.S.); yuki.yoshigae@jarmec.jp (Y.Y.); satoru.matsunaga@jarmec.jp (S.M.); 2 Laboratory of Veterinary Surgery, Faculty of Agriculture, University of Miyazaki, Miyazaki 889-2192, Japan; 3 FUJIFILM VET Systems, Tokyo 185-0013, Japan; kazumi.nibe@fujifilm.com; 4 Laboratory of Veterinary Pathology, Graduate School of Agricultural and Life Sciences, The University of Tokyo, Tokyo 113-8657, Japan; auchidak@g.ecc.u-tokyo.ac.jp

**Keywords:** laminectomy, epidural mass, inflammatory pseudotumor, dog

## Abstract

A 3-year, 9-month-old Bernese Mountain dog presented with a 10-day history of acute right hemiparesis. Neurological examination indicated abnormalities in the C1-C5 spinal segments. Magnetic resonance imaging (MRI) revealed an epidural mass at C3-C4, which was surgically removed after dorsal laminectomy. The neurological signs were completely resolved by the 12th postoperative day. The lesion was diagnosed as an inflammatory pseudotumor on histopathological examination. Another MRI performed 63 days after surgery showed no lesion regrowth, and there has been no recurrence for approximately 6 years. Reports of inflammatory pseudotumors are rare, and their treatment remains unclear. Previous reports and the course of this case suggest that a good prognosis may be achieved if complete surgical resection of an inflammatory pseudotumor arising in the epidural region can be accomplished.

## Simple Summary

Inflammatory pseudotumors are tumor-like lesions composed primarily of inflammatory cell infiltrates and fibrosis and are believed to occur in various organs throughout the body. This case report describes the diagnosis, treatment, and clinical outcome of an inflammatory pseudotumor arising epidurally in the cervical spinal cord of a dog. The dog presented with paraplegia of the right anterior and posterior limbs due to an inflammatory pseudotumor arising in the cervical spinal cord. The tumor was assessed by dorsal laminectomy and surgically removed. The dog’s clinical signs resolved quickly, and no signs of recurrence have been observed 6 years later. The preoperative and intraoperative diagnosis was difficult because magnetic resonance imaging and intraoperative cytology failed to identify an inflammatory pseudotumor. Based on the disease course in this case, we infer that the prognosis after surgical resection of inflammatory pseudotumors arising in the spinal epidural region is considered good.

## 1. Introduction

Inflammatory pseudotumors (IPTs) are non-granulomatous lesions characterized by mesenchymal cell proliferation and marked infiltration of inflammatory cells, mainly lymphocytes, plasma cells, and histiocytes. The pathogenesis of IPTs is unknown. Nevertheless, IPT is considered an immunological host response to infectious agents, microorganisms, necrotic tissue, chronic inflammation, neoplasms, or foreign bodies [1]. In humans, while IPTs usually appear in the lungs and orbits, nearly all organs have been reported as potential sites of involvement and generally have a benign course; however, early diagnosis and treatment are important because symptoms may progress and organ function may be impaired [1]. Additionally, it is rare for inflammatory pseudotumors to develop and affect the central nervous system in humans, with only a few reported cases of IPT occurring in the spinal cord [2,3,4].

In veterinary medicine, a few cases of IPTs have been reported in cats and dogs, mostly involving the orbital cavity [5,6,7,8,9], although occurrences in other organs [10,11,12,13] have been reported. The prognosis for IPT in the orbit of cats was reported to be poor because it also affected the opposite eye over time [7], although the prognosis for IPT in the bladder of dogs was good after surgical removal [13], and no common opinion on the prognosis after surgical removal of IPT in veterinary medicine has been reached.

Only five cases of IPT within the spinal canal have been reported, which include two intradural extramedullary [14,15], two epidural [16,17], and one intramedullary [18] lesions. Additionally, a case of an inflammatory myofibroblastic tumor, a type of IPT characterized by the proliferation of neoplastic myofibroblasts and considered to have malignant tumor characteristics, was described to have developed extradurally in the spinal cord [19]. The prognosis of IPT occurring within the spinal canal is also variable [14,15,16,17,18]. Herein, we report the case of a spinal epidural IPT in a 3-year, 9-month-old female Bernese Mountain dog.

## 2. Case Description

A 3-year-old female Bernese Mountain dog weighing 32.5 kg presented with a 10-day history of difficulty to stand. Neurological examination revealed acute right hemiparesis, with decreased postural responses in the right forelimb and hindlimb but normal postural responses in the left forelimb and hindlimb. Spinal segmental reflexes were normal in all four limbs. There were no findings suggestive of cranial nerve abnormalities. The neurological findings suggested localization of the lesion at the C1–C5 spinal segments. A complete blood cell count showed normal results, while serum biochemical analysis revealed mild increases in alkaline phosphatase (313 U/dL, normal range: 47–254 U/dL) and total cholesterol (327 mg/dL, normal range: 111–312 mg/dL).

**Figure 1:**
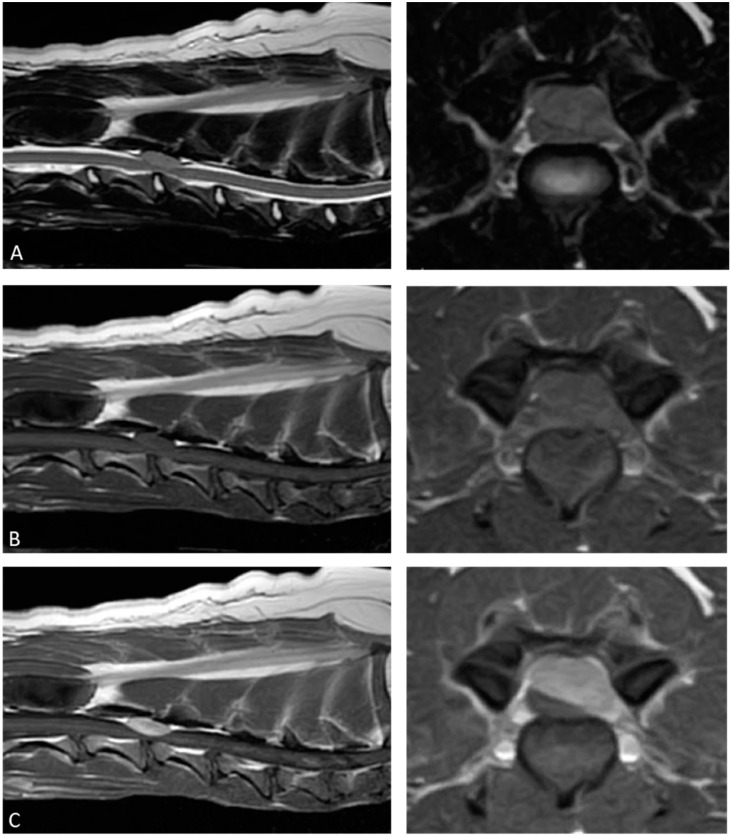
This figure is the preoperative magnetic resonance imaging of the epidural mass lesion. The mass lesion is isointense to the spinal cord parenchyma on T2-weighted images: (**A**) T1-weighted images; (**B**) the postcontrast T1-weighted images reveal a homogeneous enhancing lesion with spinal cord compression; (**C**) no abnormality is observed in the adjacent bone.

Magnetic resonance imaging (MRI; Hitachi 1.5T; Hitachi, Tokyo, Japan) revealed an epidural mass lesion measuring 22 × 12 × 7.5 mm that severely compressed the spinal cord from the right dorsal side. The lesion extended from the caudal third of the third cervical vertebra to the cranial third of the fourth cervical vertebra. Compared to the normal spinal cord, the lesion was homogenously isointense on both T2-weighted and T1-weighted images. Following intravenous administration of gadoteridol (ProHance; Bracco-Eisai, Tokyo, Japan), the lesion displayed strong, homogeneous contrast enhancement (Figure 1).

**Figure 2:**
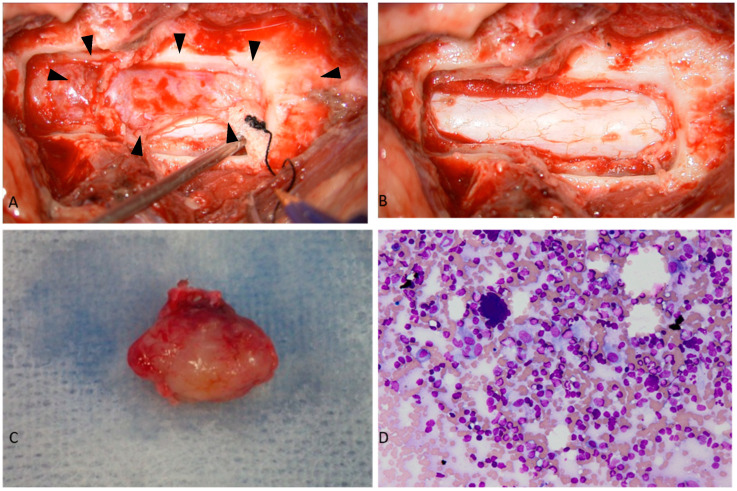
This figure shows the macroscopic findings of spinal cord lesions and intraoperative cytology findings. (**A**) A white mass lesion (inside of the arrowhead) is observed outside the spinal dura; (**B**) the appearance of the spinal cord after the excision of the lesion; (**C**) the appearance of the whole lesion; (**D**) photomicrography of the cytologic specimen revealing (Wright-Giemsa staining; magnification ×400).

Precisely 4 days after the MRI, the dog underwent dorsal laminectomy and resection of the mass lesion. During these 4 days, the patient was not taking any oral medication and was provided only cage rest. An elastic, white extradural mass was found compressing the spinal cord on removing the vertebral arches from the third to fourth cervical vertebrae (Figure 2A). The adhesion between the lesion and the dura was mild, with no continuity to the surrounding tissue observed; therefore, the lesion was exfoliated from the dura and completely removed (Figure 2B,C). An imprint cytology of the excised lesion was used for intraoperative diagnosis. Since the imprint cytology contained abundant lymphocytes, lymphoma was initially suspected (Figure 2D).

**Figure 3:**
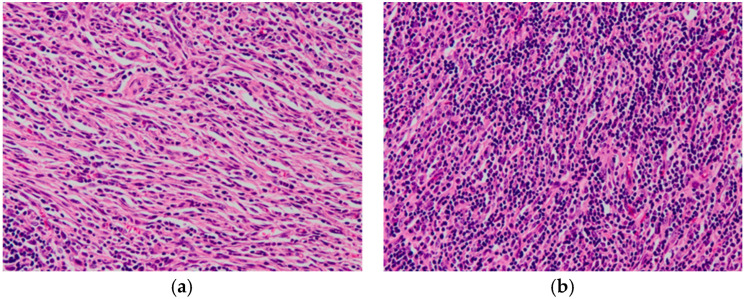
Histopathologic features of spinal mass. (**a**) Lesion with dominant proliferation of mesenchymal spindle cells with mild inflammatory cells. (**b**) Lesion with dominant infiltration by lymphocytes, plasma cells, and macrophages, (**a**,**b**) Hematoxylin and eosin staining; magnification ×400.

However, histopathological examination revealed an inflammatory tissue comprising mesenchymal spindle and inflammatory cells, mainly composed of small lymphocytes and plasma cells. Macrophages were also present in the combination. These cells showed little nuclear atypia or pleomorphism, and there were very few mitotic figures. Although the lesion did not involve spinal cord parenchyma, margin assessment was difficult due to the small tissue sample. Immunohistochemistry for Iba-1, CD3, and CD20 revealed no predominant proliferation of any particular cell type, such as T- and B-lymphocytes and histiocytes. Based on the findings, a diagnosis of IPT was made (Figure 3). Additionally, lymphocyte clonality analysis, including polymerase chain reaction for T-cell receptor and immunoglobulin genes, did not suggest monoclonal amplification of T and B cells.

**Figure 4:**
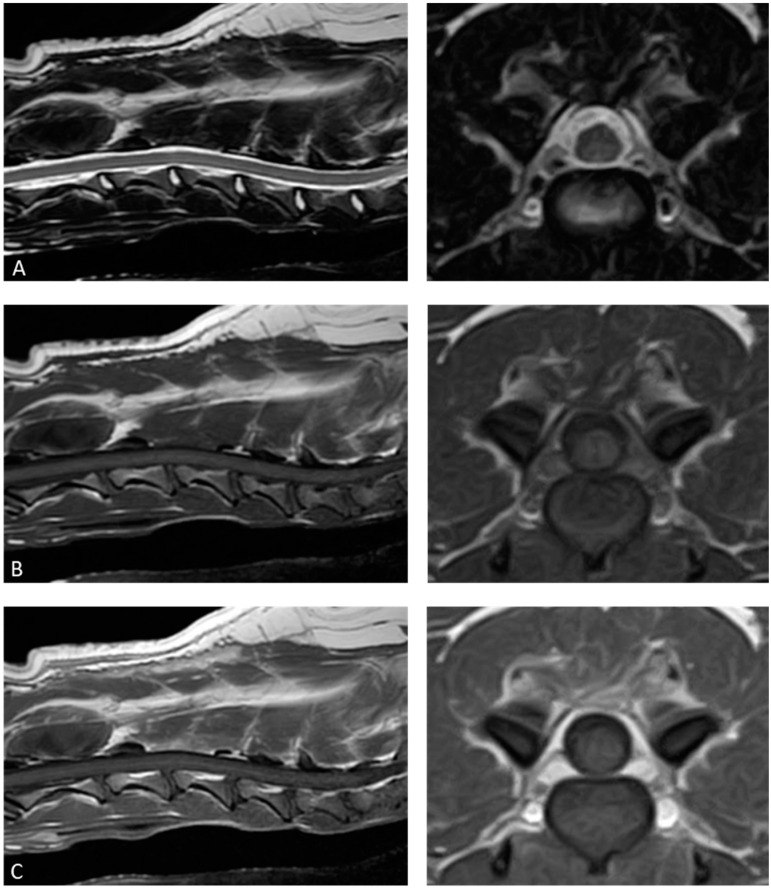
The figure shows the follow-up magnetic resonance imaging of the lesion site 63 days after surgery: (**A**) T2-weighted images; (**B**) T1-weighted images; and (**C**) T1-weighted postcontrast images. No sign of lesion recurrence is observed. The transverse images are from the level of the C3–C4 intervertebral disks.

The patient’s paralysis did not show any signs of worsening after the surgery. The dog was able to walk, and although slightly unsteady, its gait had improved compared to before surgery. As such, the patient’s postoperative progress was deemed favorable, and the dog was discharged on the fifth day after surgery. The dog received oral administration of 0.5 mg/kg prednisolone, 1 mg/kg famotidine once daily, and 20 mg/kg cephalexin twice daily for 12 days post-surgery. The clinical signs disappeared on the 12th postoperative day. Once the histopathological diagnosis was confirmed, all medications were discontinued, and no further treatment was administered. During follow-ups on the 33rd and 63rd postoperative days, no relapse of clinical signs was observed. Additionally, an MRI performed 63 days after surgery showed no recurrence of the IPT (Figure 4). On contacting the dog’s owner via telephone approximately 6 years after the surgery, we were informed that the dog’s neurological signs had remained clear.

## 3. Discussion

IPT is a benign, localized lesion characterized by mesenchymal spindle cell proliferation and infiltration of inflammatory cells, such as lymphocytes, plasma cells, and macrophages. Although a clear diagnostic criteria for IPT has not yet been obtained in the field of veterinary medicine, we made a diagnosis of IPT in this case because the histopathological findings, such as mixed inflammatory cells composed of lymphocytes and plasma cells and proliferation of spindle-shaped cells, were consistent with previous reports on IPT. IPTs are thought to be associated with trauma, postoperative inflammation, immune-autoimmune mechanisms, infections, or other malignancies, but their pathogenesis remains unclear, and no consensus exists regarding it [1,3]. In the present case, the dog had no relevant medical history, so the cause of the IPT was unknown.

In veterinary medicine, there are five reports of IPT occurring in the spinal canal. Our case was of a 3-year-old, 9-month-old Bernese Mountain dog, but previous reports have reported two cases at 1 year of age and one case each at 5, 8, and 10 years of age [14,15,16,17,18]. Consistency with respect to age of onset has not been confirmed at this time. A previous report showed that the disease had developed in the cervical spinal cord of a 1-year-old Bernese Mountain dog [17], and this case was of the same breed, with the dog developing the disease at a relatively young age of 3 years and 9 months. There may be a predisposition to developing the disease in young Bernese Mountain dogs, but further investigation is required.

Diagnostic imaging of IPTs, including computed tomography and MRI, shows various findings depending on the degree of inflammation, necrosis, fibrosis, and granulation. Previous human studies have reported no clear diagnostic imaging findings for IPTs in the spinal canal [2,20,21]. In this case, the T2-weighted images were isointense to the spinal cord, but in previous canine reports, the signal intensity on T2-weighted images varied, and consistency cannot be confirmed [14,15,16,17,18]. The findings of isointense T1-weighted images and homogeneous contrast enhancement are generally consistent with this case and previous reports in veterinary medicine, and most IPTs in the spinal canal in humans also show homogeneous contrast enhancement [2,14,15,16,17,18]. However, IPT cannot be distinguished from other tumor lesions based on MR imaging findings alone, since lymphomas, metastatic tumors, schwannomas, ependymomas, multiple myeloma, and meningiomas are similarly observed as lesions showing this homogeneous contrast enhancement. Therefore, it is difficult to make a diagnosis of IPT based on imaging findings alone without histopathologic evaluation.

In our case, IPT could not be diagnosed based on intraoperative imprint cytology. In humans, intraoperative diagnosis of IPT occurring in the spinal cord could not be made [3]. In veterinary medicine, it has also been reported that inflammatory pseudotumors of the orbit could not be diagnosed by puncture aspiration cytology [8]. Based on these findings, although continued investigation is needed, the diagnosis of IPT requires tissue resection, and it would be difficult to diagnose IPT occurring in the spinal canal pre- or intraoperatively.

Regarding IPT treatment, many studies on humans report that these lesions regress naturally. In cases where the lesions are difficult to excise because of their location, immunosuppressive therapy with steroids or cyclosporin may be adopted [1,22,23]. Natural regression has been reported as a treatment option in humans [1,4,22,23]. However, due to the unclear mechanism of spontaneous regression, surgical resection remains the first choice of treatment for IPTs in human medicine, and conservative therapy is generally not recommended [1].

In humans, surgical resection is usually recommended for IPTs in the central nervous system, with a favorable clinical course if complete resection is performed [2,3,24,25,26,27,28]. In cases where complete resection is difficult and recurrence is observed, immunosuppressive therapy using steroids or radiation therapy is selected as an adjuvant therapy to decrease the mass volume [20,24,26,29]. In previous reports, surgical resection was performed for all IPTs occurring within the canine spinal canal. The prognosis of intramedullary lesions is poor postoperatively; one case of intradural extramedullary lesion showed improvement, but neurological signs remained, and one case of postoperative worsening of symptoms resulted in euthanasia at 3 months postoperatively [14,15,18]. The prognosis of epidural lesions was excellent in this case as well as in the two previous cases [16,17]. These findings suggest that surgical resection may be effective in veterinary medicine for the treatment of IPTs in the spinal canal, especially in the epidural region, but the prognosis of intradural IPTs should be carefully monitored.

## 4. Conclusions

Although IPT should be considered a differential diagnosis for mass-like lesions within the spinal cord and spinal canal, it is difficult to diagnose an inflammatory pseudotumor on MR imaging findings or intraoperative cytology studies. Additionally, the course of the present case suggests that surgical resection of an inflammatory pseudotumor arising extradurally may have a favorable prognosis.

## Data Availability

The original contributions presented in the study are included in the article. Further inquiries can be directed to the corresponding author.

## Abbreviations

The following abbreviations are used in this manuscript:

MRI	Magnetic resonance imaging
IPT	Inflammatory pseudotumor
